# Supplementary material for: Association between Chlamydia and routine place for healthcare in the United States: NHANES 1999–2016
Source: PLoS One. 2021 May 10;16(5):e0251113. doi: 10.1371/journal.pone.0251113 (PMC8109783; doi:10.1371/journal.pone.0251113)
Supplement: Attachment — Submitted filename: Plos One Reponse to Reviewers.docx [file pone.0251113.s001.docx]

April 16, 2021

PLOS ONE

Emily Chenette, PhD

Editor-in-Chief

Sylvia Maria Bruisten, Ph.D
Academic Editor

RE: Manuscript Number PONE-D-20-16986R1
Association between Chlamydia and Routine Place for Healthcare in the United States: NHANES 1999-2016
PLOS ONE

Dear Drs. Chenette & Bruisten,

Thank you for your email dated November 25, 2020 regarding our manuscript “***Association between Chlamydia and Routine Place for Healthcare in the United States: NHANES 1999-2016***”. We greatly appreciate the suggestions and have edited our revised manuscript accordingly. Our response to the comments provided are detailed below. Please accept our revised manuscript for continued consideration for inclusion as a Research Article in *PLOS ONE*.

We appreciate your continued consideration of our work for publication in *PLOS ONE*.

Sincerely,

Cornelius D. Jamison, MD, MSPH, MS

Assistant Professor

Department of Family Medicine

University of Michigan

jcorneli@med.umich.edu

**Reviewers' Comments:**

**Reviewer #2:**

Thank you for giving me the opportunity to see a revised version of the manuscript. I appreciate the time and effort the authors put into the revisions. However, I feel that both my major comments were not addressed adequately.

1) My first major comment was about survey cycle not being included in the analyses. Although the authors did address this issue in their response (by saying it did not change the results), they did not change anything to the manuscript. I therefore encourage the authors to either included survey cycle in the main analyses in the manuscript, or as a sensitivity analyses in a supplement.

**RESPONSE:**

**We understand and appreciate this comment. We have provided the multivariate analysis that controls for the NHANES’ survey cycle as a supplement. We noted little change in the models outcome for Chlamydia in reference to the ER, from the previous OR of 1.59 (95% CI, 1.07 to 2.36) to 1.65 (95% CI, 1.10 to 2.47).**

2) My second major comment addressed possible interaction between covariates in the model (which you would expect by looking at Table 1). Performing a bivariate analyses between a covariate and CT does not address this issue. Furthermore, the authors write in their response something about “additional” models, but again, no results are shown or details are given.

**RESPONSE:**

**Thank you for this comment. In addition to performing bivariate analysis we examined the interactions and have added *p* values to Table 1 to address this concern.**

**Reviewer #3:**

I am satisfied with the authors corrections in this revised version of the manuscript and have no further comments.

**RESPONSE:**

**Thank you for your comments and the review of our revised manuscript.**
